# Role of CD133 in human embryonic stem cell proliferation and teratoma formation

**DOI:** 10.1186/s13287-020-01729-0

**Published:** 2020-05-27

**Authors:** Hua Wang, Peng Gong, Jie Li, Yudong Fu, Zhongcheng Zhou, Lin Liu

**Affiliations:** 1 State Key Laboratory of Medicinal Chemical Biology, Nankai University, Tianjin, 300071 China; 2 Department of Cell Biology and Genetics, College of Life Sciences, Nankai University, Tianjin, 300071 China

**Keywords:** CD133, Human embryonic stem cell, Teratoma

## Abstract

**Background:**

Pluripotent stem cells (PSCs), including human embryonic stem cells (hESCs), hold great potential for regenerative medicine and cell therapy. One of the major hurdles hindering the clinical development of PSC-based therapy is the potential risk of tumorigenesis. CD133 (Prominin 1, PROM1) is a transmembrane protein whose mRNA and glycosylated forms are highly expressed in many human cancer cell types. CD133 also serves as a cancer stem cell (CSC) marker associated with cancer progression and patient outcome. Interestingly, CD133 is highly expressed in hESCs as well as in human preimplantation embryos, but its function in hESCs has remained largely unknown.

**Methods:**

CD133 knockout hESC WA26 cell line was generated with CRISPR/Cas9. CD133 knockout and wide type hESC lines were subjected to pluripotency, proliferation, telomere biology, and teratoma tests; the related global changes and underlying mechanisms were further systemically analyzed by RNA-seq.

**Results:**

CD133 deficiency did not affect hESC pluripotency or in vivo differentiation into three germ layers but significantly decreased cell proliferation. RNA-seq revealed that CD133 deficiency dysregulated the p53, PI3K-Akt, AMPK, and Wnt signaling pathways. Alterations in these pathways have been implicated in tumor proliferation and apoptotic escape.

**Conclusions:**

Our data imply that CD133 could be an additional target and used as a selective marker to sort and eliminate undifferentiated cells in reducing potential teratoma formation risk of hESCs in regenerative medicine.

## Background

In regenerative medicine therapy, pluripotent stem cells (PSCs), including embryonic stem cells (ESCs) and induced pluripotent stem cells (iPSCs), exhibit unlimited self-renewal ability and pluripotency for differentiation into all cell types from all lineages in the body [1–3]. However, in cell biology, a fundamental principle is that the greater the self-renewal and pluripotency that stem cells possess, the higher the probability that they will cause tumors [4]; one major obstacle to the clinical application of these PSCs is that these stem cells and their differentiated derivatives pose cancer risks by forming teratomas [5]. Although several studies have reported methods to overcome the risk of tumorigenesis [6–8], the future of safe cell-based therapy rests on overcoming unlimited teratoma/tumor formation.

Cancer stem cells (CSCs) are capable of initiating tumor formation and can be marked by specific cell surface markers; moreover, they can initiate tumor metastasis and relapse after therapy [9–11]. Specific target of CSC marker genes may reduce tumorigenesis potential of ESCs; however, CSCs and ESCs have similarities in biomarkers, gene signatures, signaling pathways, and epigenetic regulators [12], selection of markers which will reduce tumorigenic capacity without crippling of the pluripotency and differentiation potential of ESCs may provide promising direction for safe cell-based therapy, and the cross-study may promote the design of biological and pharmaceutical tools for regenerative medicine and cancer therapies. Among CSC markers, CD133 (also known as Prominin 1, PROM1) is one of the most widely used markers for enrichment and labeling of CSCs in solid tumors [13–17]. Previous studies have focused on whether CD133 is a robust CSC marker; however, its function is still unclear in hESCs. Whether the function of CD133 is conserved in tumors and hESCs and whether CD133 is a potential target to reduce teratoma formation without radical changes to differentiation have never been systemically clarified.

We found that CD133 is highly expressed in human ESCs, and interestingly, knockout (KO) of CD133 in hESCs significantly attenuates hESC proliferation and teratoma formation but does not affect hESC pluripotency or in vivo differentiation into three germ layers.

## Materials and methods

### Cell culture

WA26 and RuES2 human embryonic cells were routinely maintained in undifferentiated state in E8 medium (A1517001, Life technologies) on Matrigel (356230, BD Bioscience)-coated tissue culture plates with daily medium feeding and passaged every 3–4 days with 0.5 mM EDTA in phosphate buffered saline (PBS) with 10 μM Rocki (sc-281642A, Santa Cruz) for maintenance. Osteosarcoma cells (U2OS) (HTB96, ATCC), cervix adenocarcinoma cells (HeLa) (CCL-2, ATCC), and human embryonic fibroblast (HEF) cells (derived from an aborted fetus) [18, 19] were cultured in high glucose Dulbecco’s modified Eagle’s media (DMEM) plus 10% fetal bovine serum (FBS) (SH30070.03, Hyclone) with 1% penicillin and streptomycin. Colon carcinoma cell line (HCT116) (CCL-247, ATCC) was cultured in RPMI1640 (11875085, Life technologies) plus 10% FBS and 1% penicillin and streptomycin. For U2OS, HeLa, HEF, and HCT116, cell culture medium was changed every 2–3 days; cells were passaged every 4–5 days with 0.25% Trypsin-EDTA (25300–072, Invitrogen) at 1:10–1:20 ratio for maintenance. All cell lines were cultured at 37 °C under 5% CO_2_.

### Knockout human PROM1/CD133 by CRISPR/Cas9 system

pSpCas9(BB)-2A-Puro (PX459) was a gift from Feng Zhang (Addgene plasmid # 48139). Guide RNA (sgRNA) of human *PROM1/CD133* was designed using the online design tool available at http://crispr.genome-engineering.org/. PX459 was digested with *Bbs*I at 37 °C for 30 min and then gel-purified according to the instructions of the gel purification kit (EG101-2, TransGene). One pair of oligos including targeting sequences was annealed and cloned into the *Bbs*I-digested PX459 vector by incubating at 25 °C for 30 min, followed by 16 °C for 30 min and hold at 4 °C. All primers used for qPCR experiments are listed in Additional file 4: Table S1.

### Transfection

hESCs (WA26) growing on matrigel-coated (Corning Bioscience) dishes with E8 medium were detached with 0.5 mM EDTA for about 6 min at 37 °C. 8 × 10^5^ cells were nucleotransfected with 10 μg of CRISPR/Cas9 plasmid using the Amaxa Nucleofector II (Lonza) and Human Stem Cell Nucleofector® Kit (Lonza) according to the manufacturer’s instructions. Nucleotransfected hESCs were plated back in a matrigel-coated dish with E8 medium supplemented with 10 μM ROCK inhibitor. CRISPR/Cas9 plasmids containing sgRNA and sgRNA-1 were transfected into HCT116 by Lipo6000 system. Cells were subjected to puromycin selection 4 h after transfection and allowed to recover for 5 days. Surviving (resistant) colonies were manually picked into new 24-well plates and then expanded for genotyping and sequencing.

### Gene expression by quantitative real-time PCR

Total RNA was isolated from cells using RNeasy mini kit (Qiagen). Two micrograms of RNA was subjected to cDNA synthesis using M-MLV Reverse Transcriptase (Invitrogen). Real-time quantitative PCR reactions were set up in duplicate with the FastStart Universal SYBR Green Master (ROX) (Roche) and run on the iCycler iQ5 2.0 Standard Edition Optical System (Bio-Rad) using primers listed in Additional file 4: Table S1. Each sample was repeated 3 times and analyzed using *GAPDH* as the internal control.

### Western blot

Western blot was performed as described previously [20] and the antibodies used were CD133 (Biorbyt, orb10288), OCT4 (Santa Cruz, sc-9081), c-MYC (Santa Cruzs, c-47694), NANOG (Santa Cruz, sc-293121), SOX2 (Millipore, AB5603), and β-actin (Abmart, P30002). Immunoreactive bands were then probed for 2 h at room temperature with the appropriate horseradish peroxidase (HRP)-conjugated secondary antibodies, anti-Rabbit IgG-HRP (GE Healthcare, NA934V), or goat anti-Mouse IgG (H + L)/HRP (ZSGB-BIO, ZB-2305). The protein bands were detected by Enhanced ECL AmershamTM prime western blotting detection reagent (GE Healthcare, RPN2232).

### Flow cytometry analysis

hESCs or HCT116 cells were collected and washed in cold PBS, and then cells were incubated with primary antibodies against CD133-APC (Miltenyi Biotec, 130-098-129) or SSEA-4-PE (BioLegend, 330,405) and incubated for 30 min on ice. Samples were washed three times with PBS and analysis was performed using a flow cytometer (BD FACS Calibur).

### Immunofluorescence

Cells were washed twice in PBS, then fixed in freshly prepared 3.7% paraformaldehyde for 15 min at 4 °C, permeabilized in 0.1% Triton X-100 in blocking solution (3% goat serum (16210064, Thermo Scientific) plus 0.5% bovine serum albumin (BSA) in PBS) for 40 min at room temperature, washed three times (each for 15 min), and left in blocking solution for 1 h. Cells were incubated overnight at 4 °C with primary antibodies against SSEA4 (MAB4304, Chemicon), OCT4 (sc-9081, Santa Cruz), and TRA-1-81 (MAB4381, Chemicon) and incubated for 1 h with secondary antibodies at room temperature. Samples were washed three times (each for 15 min) and counterstained with 0.5 μg/ml DAPI in Vectashield mounting medium. Fluorescence was detected and imaged using a Zeiss inverted fluorescence microscope.

### Cell cycle analysis

Cells were fixed in freshly prepared 70% ethanol at 4 °C overnight, then centrifuged at 1000 g for 5 min to collect cells, and stained with propidium iodide (PI) at 37 °C for 30 min in water bath. Cell cycle phases were determined by a flow cytometer (BD FACS Calibur) and the data were processed using ModFit LT.

### Telomere quantitative fluorescence in situ hybridization (q-FISH)

Telomere length and function (telomere integrity and chromosome stability) was estimated by Q-FISH as described previously [21, 22]. Cells were incubated with 0.3 μg/ml nocodazole for 4 h to enrich cells at metaphases. Chromosome spreads were made by a routine method. Metaphase-enriched cells were exposed to hypotonic treatment with 0.075 M KCl solution, fixed with methanol to glacial acetic acid (3:1), and spread onto clean slides. Telomeres were denatured at 80 °C for 3 min and hybridized with TelC-Cy3 probe (Panagene, F1002) at 0.5 μg/ml for 2.5 h at room temperature. Chromosomes were counter-stained with 0.5 μg/ml DAPI. Fluorescence from chromosomes and telomeres was digitally imaged on a Zeiss Imager Z2 microscope, using AxioCam and AxioVision software 4.6. For quantitative measurement of telomere length, telomere fluorescence intensity was integrated using the TFL-TELO program (a gift kindly provided by P. Lansdorp, Terry Fox Laboratory).

### Terminal restriction fragment (TRF) by Southern blot analysis

TRF analysis was performed as described using TeloTAGGG Telomere Length Assay Kit (12209136001, Roche). Genomic DNA from different samples was isolated with DNeasy Blood & Tissue kit (69504, Qiagen), and 1.5 μg DNA digested using *Hinf* I and *Rsa* I restriction enzymes. Digested DNA underwent electrophoresis through a 0.8% agarose gel (111860, Biowest) for 4 h at 6 V/cm in the 1 × TBE (Tris/Borate/EDTA) buffer. Gels were denatured, neutralized, and transferred to positively charged nylon membranes (RPN2020B, GE Healthcare) overnight. The membranes were hybridized in DIG Easy Hyb containing the telomere probe at 42 °C overnight. The mean TRF length was quantitatively measured according to the kit instructions.

### Telomeric repeat amplification protocol (TRAP)

Telomerase activity was determined by the TRAP method according to the manufacturer’s instructions using the TeloChaser Telomerase assay kit (T0001; MD Biotechnology, Xiamen, China). About 1 × 10^4^ cells from each sample were lysed and lysed cells heated at 70 °C for 10 min served as negative control. PCR products of cell lysates were separated on non-denaturing TBE-based 10% polyacrylamide gel electrophoresis and visualized by ethidium bromide staining.

### Teratoma test and histology by hematoxylin and eosin (H&E) staining

1 × 10^6^ hESCs were injected subcutaneously into about 6-week-old female immunodeficient nude mice. Seven mice were injected for each hESC line. Eight weeks after injection, the mice were humanely sacrificed, and the teratomas were excised; fixed in 4% paraformaldehyde at 4 °C overnight; dehydrated in gradient ethanol (70%, 85%, 95%, 100%) and xylene, the incubation time for dehydration is based on tissue size; then embedded in paraffin; and sectioned for histological examination by H&E staining.

For H&E staining, sections were deparaffinized twice in xylene (each for 5 min) and rehydrated in gradient ethanol (100%, 85%, 70%, each for 5 min), stained with hematoxylin for 4 min, washed in ddH_2_O for 5 min, treated with 1.5% hydrochloric acid-75% ethanol for 4 s, washed in ddH_2_O for 5 min followed by PBS for 2 min, then stained with eosin for about 20 s, then dehydrated in gradient ethanol (70%, 85%, 95%, 100%) and xylene, and placed in xylene and neutral resin mounting medium. All experimental procedures were processed at room temperature.

### Immunohistochemistry and fluorescence microscopy of teratoma sections

Briefly, after being deparaffinized, rehydrated, and washed in 0.01 M PBS (pH 7.2–7.4), sections were incubated with 3% H_2_O_2_ for 10 min at room temperature to block endogenous peroxidase, subjected to high pressure antigen recovery sequentially in 0.01 M citrate buffer (pH 6.0) for 3 min, incubated with blocking solution (5% goat serum and 0.1% BSA in PBS) for 2 h at room temperature, and then incubated with the diluted primary antibodies overnight at 4 °C. The following primary antibodies were used for immunocytochemistry: β-III-tubulin (CBL412, Chemicon), SMA (ab5694, Abcam), and AFP (DAK-N150130, Dako). Blocking solution without the primary antibody served as negative control. After washing with PBS three times (each for 15 min), sections were incubated with appropriate secondary antibodies at room temperature for 2 h. Then sections were washed with PBS three times (each for 15 min), and nuclei were stained with Hoechst 33342 (Sigma), placed in Vectashield mounting medium, and photographed with a Zeiss Axio Imager Z1.

### Cell apoptosis analysis

For apoptosis assays, cells were stained with Annexin V and PI using the Annexin V-FITC/PI apoptosis assay kit (Beyotime, C1062). In brief, hESC cells were digested with 0.5 mM EDTA and centrifuged, and the supernatants discarded. 1 × 10^5^ cells were washed three times with cold PBS and the supernatants discarded. The cells were then resuspended in 195 μl binding buffer from the kit. The cells were then incubated with 5 μl Annexin V-FITC and 10 μl propidium iodide for 20 min in the dark at 25 °C, and then the percentage of apoptotic cells was detected by a flow cytometer (BD FACS Calibur).

### RNA-sequencing and analysis

Total RNA was extracted from cells (at passage 20) using RNeasy mini kit (Qiagen) according to the manufacturer’s instructions. Sequencing libraries were generated using Smart-seq2 protocol [23] and index codes were added to attribute sequences to each sample. After cluster generation, the library preparations and sequenced on Illumina platform and 150 bp paired-end reads were generated. The RNA-seq reads were aligned to the human reference genome hg19 using Hisat2. Prior to differential gene expression analysis, the read counts were adjusted for each sequenced library by *featureCounts* R package through one scaling normalized factor. Differential expression analysis of two conditions was performed using the *DESeq2* R package. All differentially expressed genes were determined by fold change > 1.5 and *q* value < 0.05.

Functional enrichment (Kyoto Encyclopedia of Genes and Genomes, KEGG) of gene sets with different expression patterns was performed using *clusterProfiler* [24]. The heat maps were drawn by the function “pheatmap” of R packages *pheatmap* and correlation coefficients were calculation by the function “cor” in *R*. Scatter plots were generated using the ggplot2 package to graphically reveal genes that differ significantly between two samples. Gene expression data were analyzed using Gene Set Enrichment Analysis (GSEA) [25, 26] module in GenePattern. Metabolic interaction network was analyzed by Cytoscape plug-in BinGO.

### Rescue of CD133

The CD133 overexpression plasmid was constructed by subcloning WA26 wide type (WT) CD133 cDNA with primers listed in Additional file 4: Table S1, and cloned into PB-CAG-FLAG overexpression vectors at the AscI-BamHI site. All cDNA cloning events were confirmed by restriction digest (BamHI, R0136V, NEB; AscI, R0558V, NEB), and digestion products with right size were confirmed by sequencing analysis. The PB-CAG-FLAG plasmid with green fluorescent protein (GFP) sequence served as vehicle control. PB-CAG-FLAG-GFP control and PB-CAG-FLAG-CD133 overexpression plasmid were nucleotransfected into WA26 CD133 WT and KO cells using Human Stem Cell Nucleofector® Kit (Lonza) according to the manufacturer’s instructions. Nucleotransfected hESCs were plated back in a matrigel-coated dish with E8 medium supplemented with 10 μM ROCK inhibitor. hESCs were subjected to hygromycin selection 4 h after nucleofection and allowed to recover for 5 days. Surviving colonies were manually picked into new 24-well plates coated with matrigel with E8 and then expanded for further analysis.

### Analysis of potential off-target sites via DNA sequencing

The potential off-target sites (OTs) were provided by crispor.tefor.net/crispor.py website according to previously published protocol [27]. The top six potential OTs were scored and another three sites which located at the exon region and with cut-frequency determination (CFD) off-target scores > 0.02 were selected for off-target detection. The potential OT regions were analyzed by PCR and then sent for Sanger sequencing with specific primers (Additional file 4: Table S1).

### Statistical analysis

Correlation between telomere length and expression level of gene was examined using Pearson’s correlation coefficient. All results were analyzed by student’s *t* test and the resulting *P* values were shown. Significant differences were defined as *P < 0.05, **P < 0.01, and ***P < 0.001. The results were shown as mean ± SD.

## Results

### CD133 expression is elevated in hESCs

**Fig. 1:**
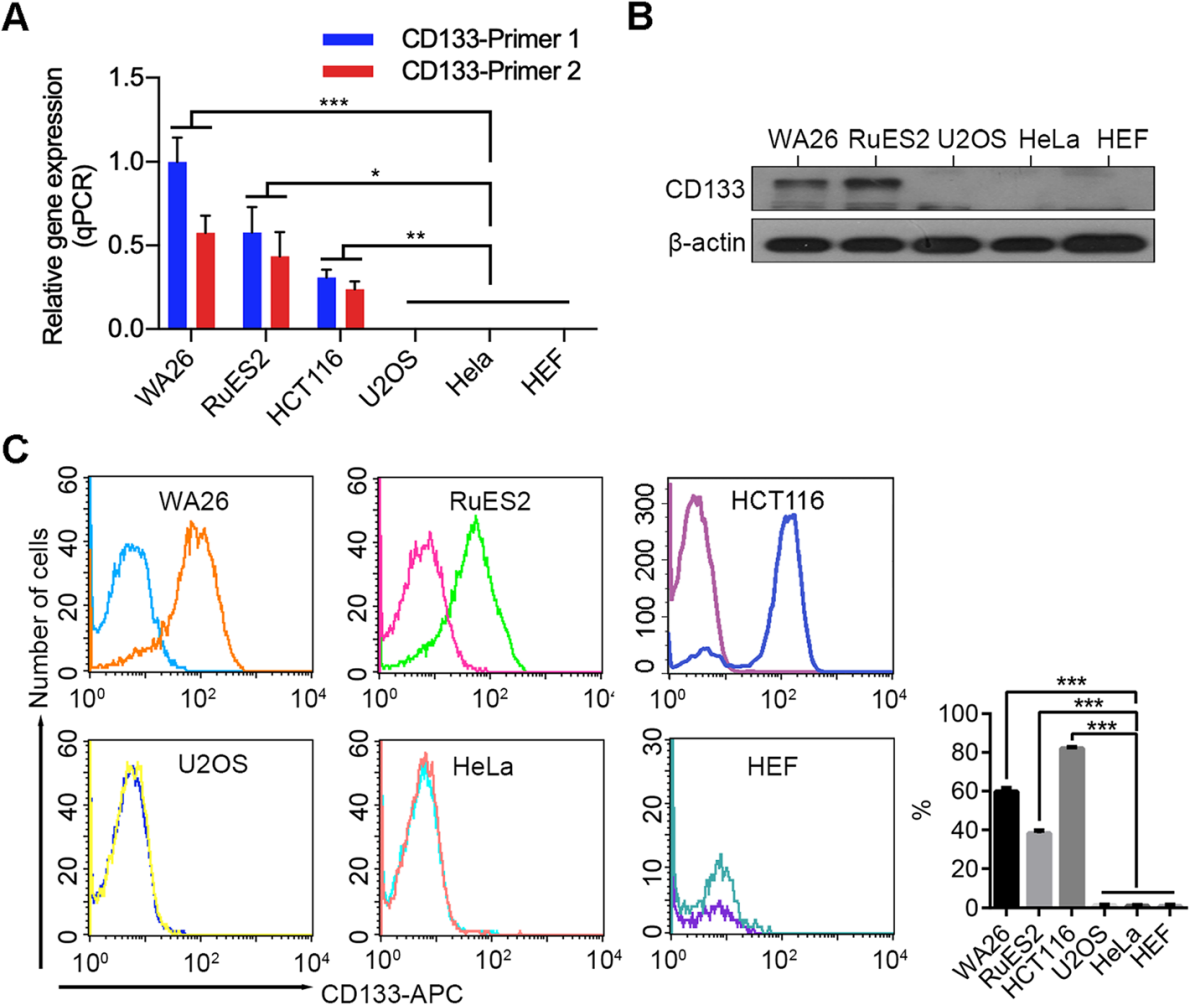
CD133 is highly expressed in human embryonic stem cells. **a** Analysis of *CD133* mRNA expression levels of two human embryonic stem cell (WA26 and RuES2, passage 20), HCT116, U2OS, HeLa, and HEF by RT-qPCR. Two paired primers were used. Bars indicate mean ± SD (*n* ≥ 2). **b** The protein level of CD133 in human cell lines determined by Western blot (immunoblotting) using a commercial CD133 antibody (Biorbyt, orb10288). **c** Flow cytometry analysis of these human cells using a commercial anti-CD133/1 (AC133)-APC conjugated antibody (Miltenyi Biotec, 130-098-129). The bar plot shows the percentage of CD133-positive cell in each cell line

Based on our single cell analysis, hESCs express CD133 at high levels [18]. To validate the high expression of CD133 in hESCs, we assessed CD133 expression by qPCR using two paired primers (different loci at the cDNA of *PROM1*) in six human cell lines, including two hESC lines (WA26 and RuES2), a colon adenocarcinoma cell line (HCT116), an osteosarcoma cell line (U2OS), a cervix adenocarcinoma cell line (HeLa), and a human embryonic fibroblast (HEF) cell line (Fig. 1a). We found that both hESC cell lines (WA26 and RuES2) expressed higher levels of *CD133* mRNA than the other three human cell lines (U2OS, HeLa, and HEF) by using two pairs of primers for CD133 (Fig. 1a). We confirmed the qPCR data by performing Western blot analysis on these cell lines, and the data also showed that CD133 was specifically expressed in hESCs (Fig. 1b). In addition, quantification of the CD133-positive cell proportion using a widely used allophycocyanin (APC)-conjugated CD133 antibody by flow cytometry also revealed high expression of CD133 in hESCs and HCT116 cells, whereas no obvious CD133-positive cells were found in U2OS, HeLa, or HEF cells (Fig. 1c). These data indicate that there is a strong correlation between CD133 and the pluripotency of stem cells.

### CD133 deficiency does not affect pluripotency gene expression in hESCs

**Fig. 2:**
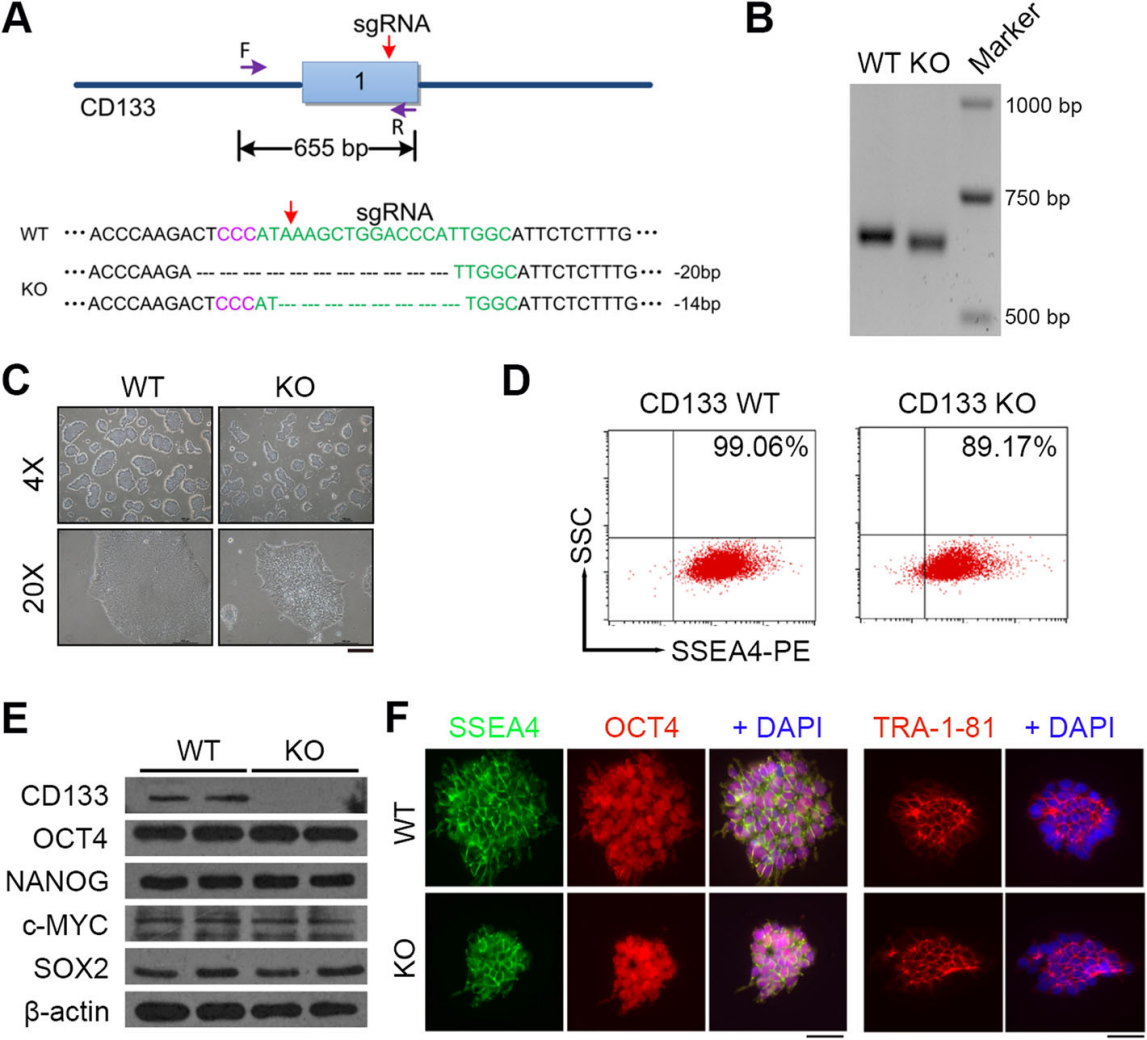
Knockout (KO) of CD133 in WA26 hESCs by CRISPR/Cas9. **a** Schematic diagram of the strategy for generating a *CD133* knockout hESC line by CRISPR/Cas9. **b** PCR amplification of the targeted region in the deletion mutants. **c** Colony morphology of *CD133* KO and WT hESCs. Scale bar = 100 μm. **d** Analysis of SSEA4+ cells in *CD133* KO and WT hESCs by flow cytometry. **e** Western blot analysis of protein expression of CD133 and pluripotency markers in *CD133* KO and WT hESCs. Cell lysates were collected and analyzed by immunoblotting using the indicated antibodies. **f** Immunofluorescence staining of SSEA4, OCT4 and TRA-1-81 in *CD133* KO, and WT hESCs. Scale bar = 20 μm. ESCs were at passage 27

To explore the roles of PROM1/CD133 in hESCs (WA26), we deleted the gene with the CRISPR/Cas9 system. We originally designed a Cas9 sgRNA targeting exon 1 of *PROM1/CD133* and used nucleofection to transfect cells with the Cas9 plasmid with high transfection efficiency (Fig. 2a; Additional file 4: Table S1). Ultimately, we selected a knockout (KO) clone with a small deletion frame shift band for further study (Fig. 2b). This clone (KO) was further confirmed by Sanger sequencing to have a 20-bp/14-bp deletion within exon 1 of *PROM1/CD133* (Fig. 2a). To determine whether the CD133 protein had been disrupted, cells from WT and KO hESCs were used for Western blot analysis, and the data showed that CD133 protein was only detected in WT cells, while it was disrupted in KO hESCs (Fig. 2e). To exclude potential off-target effects, we tested nine sites that are highly homologous to on-target sites in the human genome (Additional file 5: Table S2) and found no off-target effects in CD133 KO hESCs by Sanger sequencing. After stabilizing knockout of CD133 in hESCs, CD133 KO cells showed smaller clones than WT cells (Fig. 2c). The KO cells showed decreased expression of the pluripotency stem cell marker SSEA4 (Fig. 2d). The loss of CD133 in KO hESCs was validated by Western blot; however, the loss of CD133 in hESCs did not influence pluripotency markers including OCT4, NANOG, c-MYC, and SOX2 (Fig. 2e). Immunofluorescence staining of the stem cell markers OCT4 and TRA-1-81 also confirmed no obvious difference between WT and KO hESCs (Fig. 2f). These data indicated that we successfully established the CD133 KO hESC cell line and that disturbance of CD133 in hESCs does not directly influence the pluripotency of hESCs.

### CD133 deficiency dysregulates cell proliferation but not the in vivo three-germ layer differentiation ability of hESCs

**Fig. 3:**
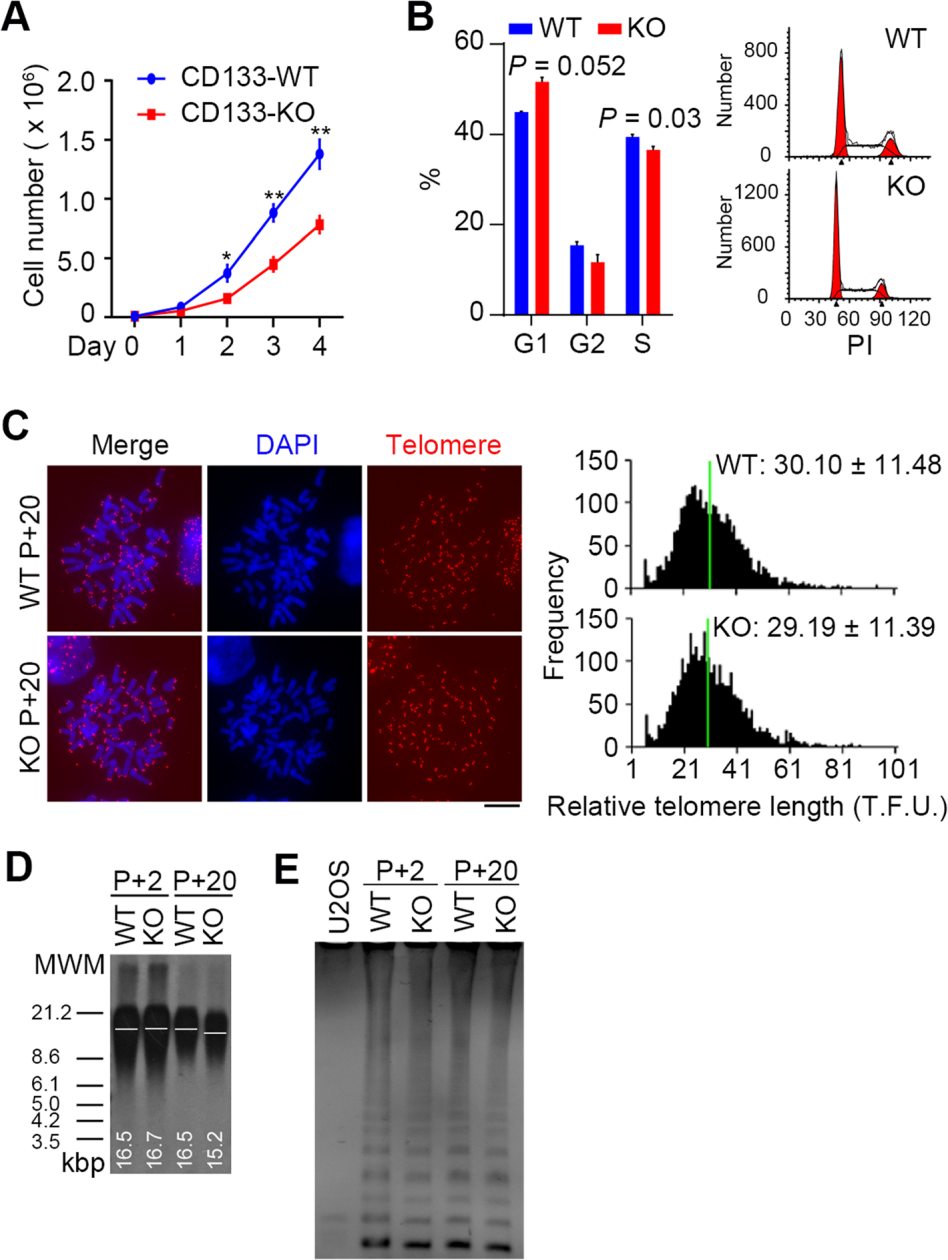
CD133 deficiency represses cell growth of hESCs. **a** Growth curves show that CD133 KO significantly restrains hESC proliferation. Bars indicate mean ± SD (*n* = 3). **b** Cell cycle analysis of *CD133* KO hESC by flow cytometry. The peaks in the illustration correspond to the G1/G0, S, and G2/M phases of the cell cycle. Histogram showing the percentages of cells in each phase of the cell cycle. Bars indicate mean ± SD (*n* = 3). **c** Representative telomere Q-FISH images of *CD133* KO and WT WA26 hESCs. Telomeres are labeled by CY3 probes (red), and chromosomes are labeled with DAPI (blue). Scale bar = 10 μm. Histogram shows distribution of relative telomere length expressed as fluorescence intensity (TFU, telomere fluorescence unit) by telomere Q-FISH analysis. Green line indicates median telomere length. Average telomere length is shown as mean TFU ± SD. **d** Telomere restriction fragment (TRF) analysis the telomere lengths in *CD133* KO hESC compared with WT ESCs at different cell passage (passage 2 vs. passage 20). **e** Telomerase activity by TRAP assay of *CD133* WT and KO hESCs

To elucidate the effect of CD133 deficiency on the cell growth of hESCs, we examined the growth curves of KO and WT hESCs. The data showed that KO cells displayed lower growth rates than WT cells (Fig. 3a), and this likely contributed to smaller clones of CD133 KO cells (Fig. 2c). Additionally, as CD133 has been widely studied in colon adenocarcinoma cell lines [28–30], including the HCT116 line, we introduced CD133 knockout in the colon carcinoma cell line HCT116 with the CRISPR/Cas9 system. The sgRNA used for WA26 cells and another sgRNA (sgRNA-1) that also targeted exon 1 of PROM1/CD133 were transfected into HCT116 cells. Finally, a clone transfected with the sgRNA-1 Cas9 plasmid was determined to exhibit a 152-bp insertion between the 10th and the 11th base of the CDS region by Sanger sequencing (Additional file 1: Figure S1A), and the CD133 protein was confirmed to be detectable in WT but disrupted in KO HCT116 cells by flow cytometry analysis (Additional file 1: Figure S1B). HCT116 cells with deletion of CD133 displayed abnormal morphology (Additional file 1: Figure S1C) and showed significantly reduced cell proliferation (Additional file 1: Figure S1D), consistent with our hESC data and previous reports [28–30]. To further validate that CD133 KO was the direct cause of the impaired proliferation of hESCs, we overexpressed CD133 in CD133 KO cells, which rescued the proliferation disturbance caused by CRISPR/Cas9 KO under the same conditions (Additional file 2: Figure S2).

The cell cycle was analyzed immediately after cell proliferation using propidium iodide staining, and flow cytometry revealed that compared to WT cells, KO cells showed a significantly decreased population in S phase (*P* = 0.03), whereas the cell population in G1 phase was increased (Fig. 3b).

Telomere length can be considered a biological marker for cell proliferation, and telomere length maintenance is also a very important feature of ESCs [31, 32]. We found a slight difference in telomere length in the KO cells compared with the WT cells by qFISH (Fig. 3c), and confirmed the finding with telomere terminal restriction fragment (TRF) analysis (Fig. 3d). KO hESC telomeres became 1 kilobase pair shorter after many passages. However, we did not observe obvious changes in telomerase activity through TRAP assay despite the passages (Fig. 3e).

**Fig. 4:**
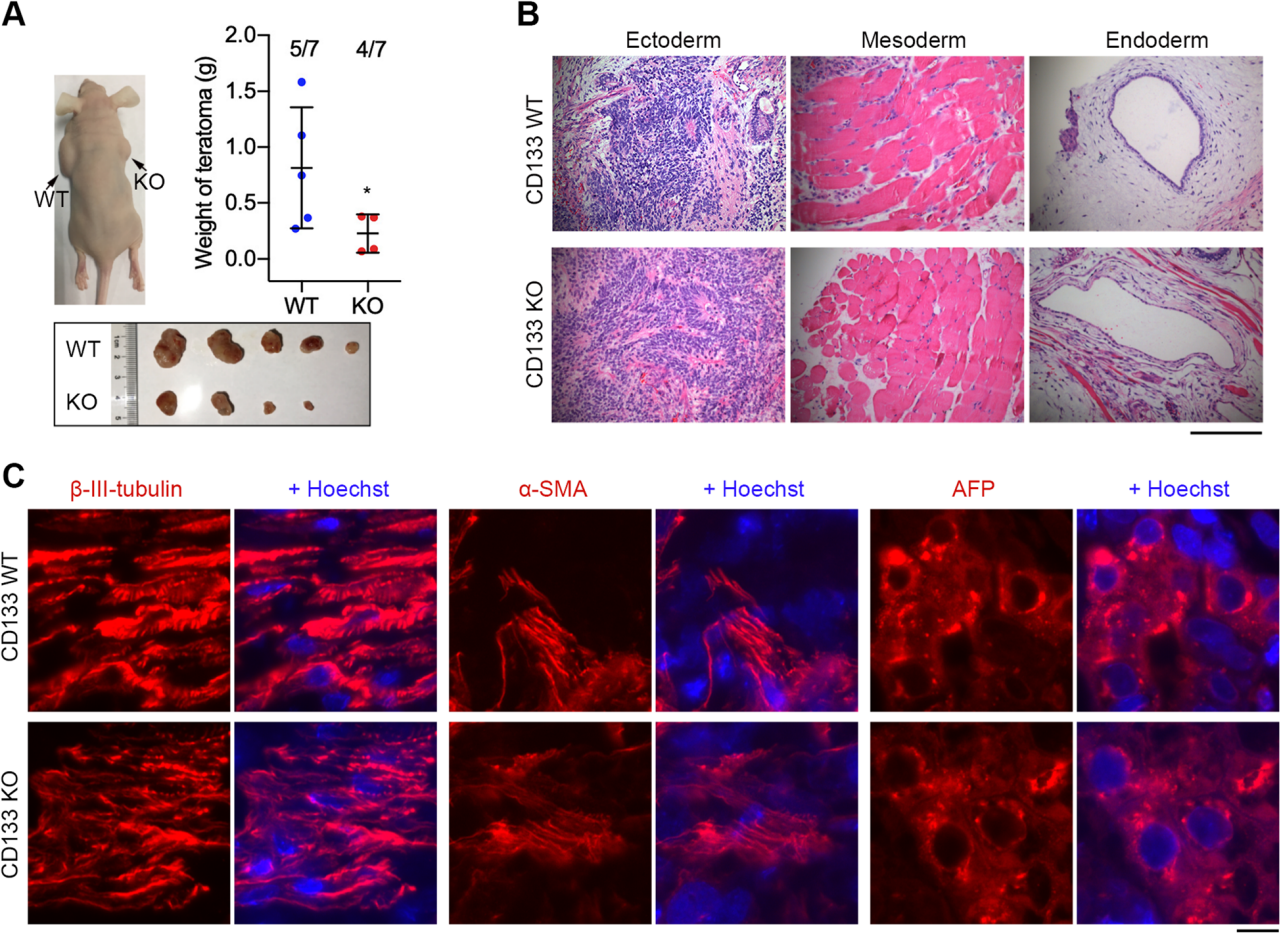
CD133-deficient hESCs maintain differentiation capacity into three germ layers. **a** Differentiation in vivo of *CD133* KO and WT hESCs (passage 20) by teratoma formation test following injection into nude mice. Black arrows indicate teratomas on the back of nude mice. **b** Hematoxylin and eosin staining of teratoma tissues derived from *CD133* KO and WT hESCs. All teratomas consist of representative derivatives of three germ layers, including epidermis (ectoderm), cartilage (mesoderm), and gland epithelium (endoderm). Scale bar = 200 μm. **c** Immunofluorescence of the teratomas showing markers representative of three germ layers, beta-III-tubulin (ectoderm), smooth muscle actin (SMA, mesoderm), and alpha 1-fetoprotein (AFP, endoderm). Scale bar = 10 μm

In vivo differentiation was tested by analysis of teratoma formation following transplantation of CD133 KO and WT hESCs into nude mice. The macroscopic teratomas were observed in nude mice after 4 weeks with injection of WT hESCs; however, it was observed after 6 weeks in the mice with injection of KO hESCs. After 8 weeks, five teratomas were formed from seven injection sites in the WT group, and four smaller teratomas in the KO group (Fig. 4a). The size and weight of teratomas derived from WT hESCs were larger and significantly heavier than those of KO hESCs (*P* = 0.0391) (Fig. 4a). Although KO and WT hESCs showed difference at teratoma size and weight, KO and WT hESCs both formed teratomas and could differentiate into epidermal (ectodermal), cartilaginous (mesodermal), and glandular epithelial (endodermal) cell layers, as determined by hematoxylin-eosin (HE) staining (Fig. 4b). Immunofluorescence staining of markers for mesoderm (alpha smooth muscle, α-SMA), ectoderm (β-III-tubulin), and endoderm (alpha fetoprotein, AFP) also indicated that both WT and KO hESCs had the ability to differentiate into all three embryonic germ layers (Fig. 4c). These data suggest that CD133 KO hESCs may have the potential for safe transplantation while maintaining the ability to differentiate into three germ layers.

### Loss of CD133 decreases cell malignancy, as suggested by RNA sequencing

**Fig. 5:**
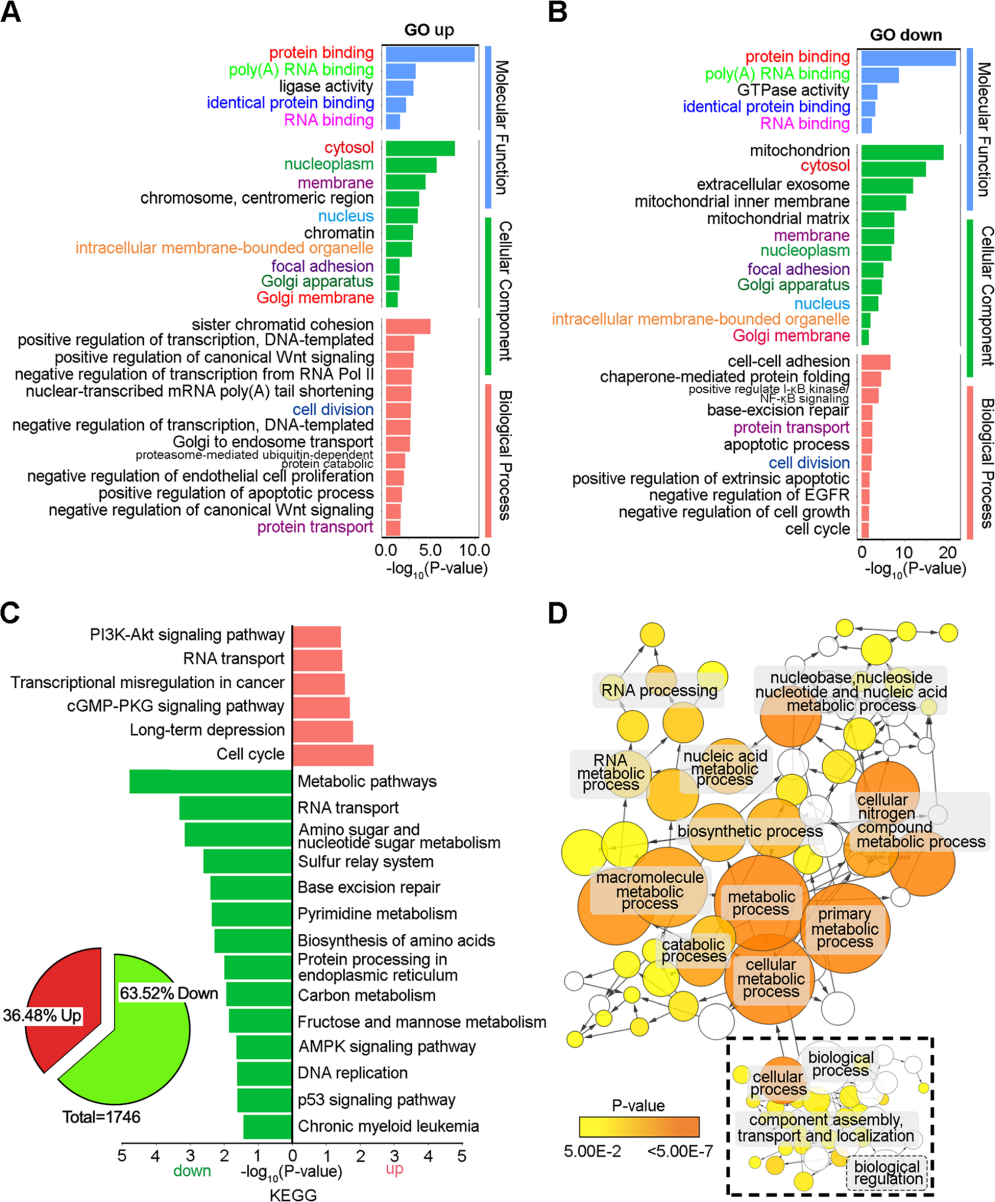
Global gene expression profiling of CD133 KO and WT hESCs by RNA-seq. **a**, **b** Significant gene ontology (GO) analyses of differentially expressed genes in *CD133* KO hESCs compared with WT hESCs (passage 20) (fold change ≥ 1.5). **c** KEGG pathway enrichment analysis of differential expression genes in *CD133* KO versus WT hESCs. Left panel shows percentage of globally down- and upregulated genes. *Y*-axis shows the pathway category and *X*-axis shows the negative logarithm of the *P* value (−LgP). A larger –LgP indicate a smaller *P* value for the difference. **d** Interaction network using Cytoscape plug-in BinGO indicates main downregulated metabolic process. Yellow nodes: nodes with *P* value < 0.05 and multiple testing corrected by Benjamini and Hochberg false discovery rate (FDR) correction. Dotted box indicates other biological processes that potentially regulate the metabolic process

**Fig. 6:**
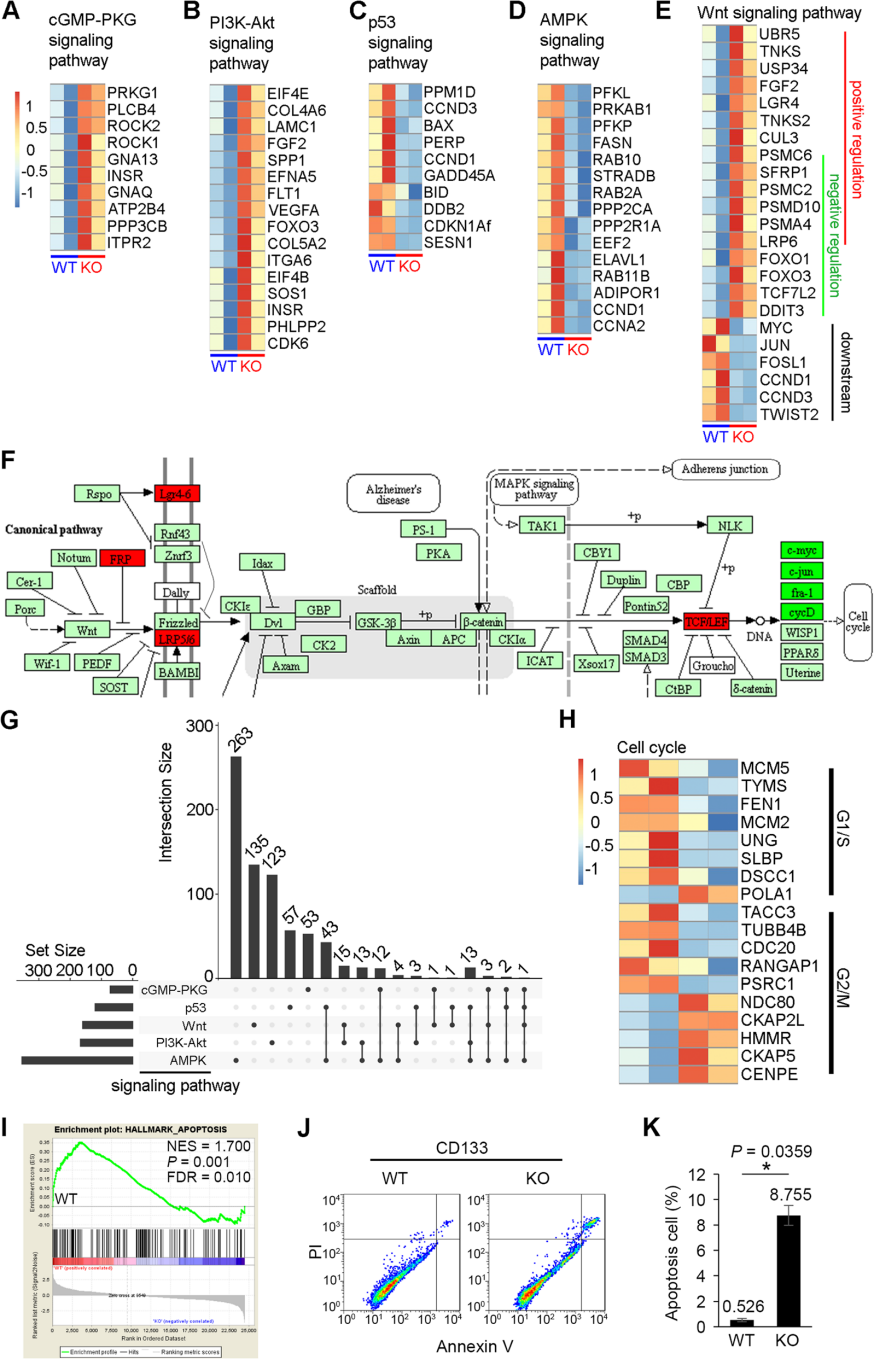
Dysregulated pathways involved in cell proliferation and apoptosis. **a**–**e** Heatmap of differentially expressed genes enriched in cGMP-PKG (**a**), PI3K-Akt (**b**), p53 (**c**), AMPK (**d**), and Wnt (**e**) signaling pathways. **f** Down- (dark green) and up- (red) regulated genes involved in Wnt signaling pathways. **g** Gene overlap analysis among cGMP-PKG, PI3K-Akt, p53, AMPK, and Wnt signaling pathways. *X*-axis “Set Size” indicates total gene number for each KEGG signaling pathway, *Y*-axis “Intersection Size” indicates total overlapped gene number between the indicated pathway below (single point) with all other pathways, or two different signaling pathways below (two points connected by one line). **h** Heatmap of differentially expressed genes regulating cell cycle. **i** GSEA enrichment plots showing that loss of CD133 in hESCs results in dysregulation of apoptosis signaling. **j** Analysis of apoptotic cells in each group detected by flow cytometry following staining with the Annexin V-FITC and PI. **k** Percentage of cells in different groups of apoptosis displayed in histograms and as mean ± SD

To define the global gene expression signature of CD133, we performed RNA sequencing (RNA-seq) of our CD133 KO and WT hESCs at passage + 20. Analysis of the significantly enriched Gene Ontology (GO) terms showed that many terms were shared between up- and downregulated genes, such as the protein binding, RNA binding, cytosol, and membrane terms, indicating global dysregulation (Fig. 5a, b). As CD133 is a cholesterol-interacting pentaspan transmembrane glycoprotein, we speculated that the destruction of CD133 may disturb some basic biological processes and related signal transmission, which was also demonstrated by changes in protein transport, cell division, cell proliferation, apoptosis, and other processes (Fig. 5a, b). To further glean biological insight from the transcript-level responses to loss of CD133, we performed Kyoto Encyclopedia of Genes and Genomes (KEGG) enrichment analysis to explore functional pathways enriched for the differentially expressed genes (DEGs) between KO and WT cells. Among the 1746 differentially expressed genes, 63.52% were downregulated, and most of these downregulated genes were associated with metabolic pathways (Fig. 5c) and metabolic changes, including changes in biosynthetic processes, catabolic processes, cellular nitrogen compound metabolic processes, nucleic acid metabolic processes, and other processes (Fig. 5d), coinciding with decreased proliferation. According to the interaction network, the metabolic changes were closely related to cellular biological regulation and were also accompanied by dysregulation of essential signaling pathways, such as the PI3K-Akt, AMPK, and p53 pathways (Fig. 5c). The significantly changed genes enriched in these KEGG pathways are shown in heatmaps (Fig. 6a–e). Although the upregulated genes were enriched for the cGMP-PKG and PI3K-Akt signaling pathways, the downregulated genes were enriched for the AMPK signaling pathway (Fig. 6a–d), and the downstream changes were not simply corresponding upregulation or downregulation. Taking Wnt signaling as an example, there were genes enriched both for positive and negative regulation of the Wnt signaling pathway; however, the downstream genes of the Wnt signaling pathway were mostly downregulated (Fig. 6e, f). Overall, the final changes after CD133 deletion resulted from coregulation of all those pathways, and these pathways also interacted with each other not only on the regulation level but also on the gene level (Fig. 6g). The downstream changes in the cell cycle were also consistent with changes in the S phase population of CD133 KO hESCs (Fig. 3b), and significant DEGs associated with the cell cycle were mostly downregulated (Fig. 6h). Notably, neither GO/KEGG terms nor specific genes were found to be significantly related to hESC pluripotency or three-germ layer differentiation (Additional file 6: Table S3; Additional file 7: Table S4), consistent with our results described above.

To obtain an overview of global changes, we subjected all of the dysregulated genes to KEGG analysis, and the results showed substantial enrichment for pathways in cancer (hsa05200). We also found the involvement of the Wnt, PI3K, MAPK p53, cell cycle, and apoptosis pathways, which are fundamental for tumorigenesis. This result confirmed the findings of disturbance in both proliferation and apoptotic evasion after CD133 KO (Additional file 3: Figure S3A) and was consistent with reduced cell proliferation and inhibited teratoma formation. Apoptosis is a process of programmed cell death, and the tumor protein p53 is a nuclear transcription factor that regulates the expression of a wide variety of genes involved in apoptosis and growth arrest in response to genotoxic or cellular stress. The p53 tumor suppressor protects against cancer by eliminating cells that have suffered DNA damage or that proliferate in an uncontrolled manner by inducing apoptosis [33–36]. Gene Set Enrichment Analysis (GSEA) also revealed most of the differentially expressed genes to be known specific hallmarks of apoptosis (Fig. 6i). To investigate whether apoptosis was involved in the effects of CD133 deficiency, we determined the proportion of apoptotic cells using a flow cytometer by double staining of cultures with propidium iodide (PI) and annexin V-FITC (Fig. 6j; Additional file 3: Figure S3B). The results showed that fewer apoptotic cells were found among WT hESCs (average 0.526%); however, the proportion of late apoptotic cells was increased significantly among KO hESCs (average 8.755%) compared with WT hESCs (*P* = 0.0359) (Fig. 6k). These results confirmed that loss of CD133 resulted in the evasion of apoptosis.

## Discussion

CD133 is a transmembrane protein whose mRNA and glycosylated form are extensively highly expressed in many human cancer cells and hESCs. CD133 has been extensively utilized to enrich CSCs from human solid tumors along with other stem cell markers [37–42]. CD133 is not only a cell surface marker; in human cancers, it has been demonstrated to regulate tumorigenesis, cell self-renewal, and angiogenesis and to promote tumor metastasis and cancer cell migration [29, 43–45]. We (in this study) and others [46–49] have found that the high expression of both the mRNA and protein of CD133 in hESCs is similar to that in many human cancer cells, and here we have characterized the role of CD133 in hESCs using CRISPR/Cas9 and RNA sequencing.

Numerous studies have investigated the mechanisms of CD133 involvement in cancer. The CD133 protein can physically associate with HDAC6 and β-catenin in a ternary complex to regulate the Wnt/β-catenin signaling pathway [29]. The CD133-p85 interaction can activate the PI3K/Akt pathway to promote tumorigenesis [45]. The CD133-associated CD90-integrin-mTOR/AMPK-CD133 signaling axis is also critical for promoting liver carcinogenesis [50]. Our RNA-seq results, albeit on the transcriptional level, also showed changes in the above signaling pathways, suggesting the disturbance of related protein interactions after CD133 deletion and the conserved functions of CD133 in tumor cells and ESCs. CD133 expression levels are correlated with the cell cycle DNA profiles of colon cancer cells, melanoma cells, and hESCs [47]. We also found that CD133 KO hESCs showed lower proliferation levels and dysregulated cell cycles compared with those of CD133 WT hESCs. Telomere length maintenance is critical for unlimited self-renewal and pluripotency [51]. But telomere length and telomerase activity do not differ between WT and CD133 KO hESCs, suggesting that telomere function does not explain the defects of CD133-deficient cells.

Concerns about potential tumorigenicity limit ESC-based cell therapy. Two previous studies have investigated CD133+ and CD133− populations in H9 hESC line; however, they obtained contradictory results regarding the three-germ layer differentiation potential of embryoid bodies (EB) [52, 53], raising suspicion about differences in the in vitro culture and differentiation systems. Among the established techniques for the pre-clinical safety assessment of PSCs, the teratoma assay not only measures differentiation but also allows insight into a PSC’s malignant potential [54]. Notably, one of the above studies regarding CD133 in hESCs also carried out the teratoma formation assay [53], and consistent with our observations, CD133- hESCs were able to differentiate into three germ layers. Interestingly, the authors showed that CD133+ cells gave rise solely to ectoderm, indicating the contribution of these cells to neural differentiation, consistent with our KEGG results that the upregulated genes in CD133 KO hESCs enriched for long-term depression (*P* value = 0.0234) and Alzheimer’s disease (*P* value = 0.0888, data not shown). This point cannot be ignored in the context of clinical application. However, it is promising that there are no substantial changes in the potential to differentiate into the three germ layers, indicating fundamental value for regenerative medicine.

Overall, our study reveals the role of CD133 in hESCs and demonstrates that cell surface marker CD133 could be a target to reduce possible teratoma formation of hESCs. Selective elimination of undifferentiated human PSCs used for regenerative medicine has been proposed to inhibit tumor formation in vivo [55, 56]. Our data suggests that CD133 as a robust cell surface marker could be elegantly employed to sort out and delete the undifferentiated ESCs in the potential clinic application after directed lineage specific differentiation of hESCs.

## Conclusions

In summary, we report that CD133 deficiency does not affect hESC pluripotency or in vivo differentiation into three germ layers but significantly decreases cell proliferation. Moreover, CD133 deficiency dysregulates the p53, PI3K-Akt, AMPK, and Wnt signaling pathways, which is implicated in malignant proliferation and apoptotic failure. Our data support possible application of CD133 as a selective marker to sort and eliminate undifferentiated cells in reducing potential teratoma formation risk of hESCs in regenerative medicine.

## Supplementary information

**Additional file 1: Figure S1.** Knockout of *CD133* in HCT116 by CRISPR/Cas9. **(A):** Sequencing results show a 152bp insertion between the 10th and the 11th base of CDS induced by CRISPR/Cas9 in HCT116 KO clone. **(B):** Flow cytometry analysis of relative protein level of CD133 in HCT116 WT and KO cell lines. **(C):** Morphology of *CD133* WT and KO HCT116 cells. Scale bar = 50 μm. **(D):** Growth curves of 7 days show that *CD133* KO significantly restrains HCT116 proliferation. Bars indicate mean ± SD (*n* = 6).

**Additional file 2: Figure S2**. Rescue of *CD133* in *CD133* knockout WA26 hESCs. (**A**)**:** Relative protein level of CD133 and GFP in human cell lines determined by Flow cytometry analysis. (**B**)**:** Analysis of *CD133* mRNA expression of indicated cell lines by RT-qPCR with CD133-qF/R primers (Additional file 4: Table S1). Bars indicate mean ± SD (*n*≥2). (**C**)**:***CD133* overexpression significantly rescues the *CD133* KO hESC proliferation phenotype by counting cell number. Bars indicate mean ± SD (*n* = 4).

**Additional file 3: Figure S3**. Overview of general pathways in cancer. (**A**)**:** All down (dark green) and up (red) regulated genes involved in KEGG cancer pathways, suggesting dysregulation of apoptosis and proliferation. Data analyzed by KEGG Mapper-Search & Color Pathway website. (**B**)**:** Isotype controls for apoptotic analysis in Fig. 6j.

**Additional file 4: Table S1.** Primers for CAS9, over expression and gene expression analysis.

**Additional file 5: Table S2.** Potential off-target sites (OTs) and primers.

**Additional file 6: Table S3.** Pluripotency genes from RNA-seq.

**Additional file 7: Table S4.** Genes related to embryonic germ layers from RNA-seq.

## Data Availability

The data and materials supporting the findings of this study are available within the article or its supplementary materials. The RNA-seq raw data have been deposited on GEO (Gene Expression Omnibus) under accession number GSE140350. The datasets used and/or analyzed during the current study are available from the corresponding author on reasonable request.

## Abbreviations

PSCs: Pluripotent stem cells
hESC: Human embryonic stem cell
iPSC: Induced pluripotent stem cell
FBS: Fetal bovine serum
PSCs: Pluripotent stem cells
CSC: Cancer stem cell
HEF: Human embryonic fibroblast
PBS: Phosphate buffered saline
BSA: Bovine serum albumin
DMEM: Dulbecco’s modified Eagle’s media
FACS: Fluorescence-activated cell sorting
GFP: Green fluorescent protein
PI: Propidium iodide
q-FISH: Quantitative fluorescence in situ hybridization
TFU: Telomere fluorescence unit
TRF: Terminal restriction fragment
OTs: Off-target sites
TBE: Tris/Borate/EDTA
TRAP: Telomeric repeat amplification protocol
CFD: Cut-frequency determination
ICMs: Inner cell masses
APC: Allophycocyanin
KO: Knockout
GO: Gene Ontology
WT: Wide type
KEGG: Kyoto Encyclopedia of Genes and Genomes
DEGs: Differentially expressed genes
GSEA: Gene Set Enrichment Analysis
FDR: False discovery rate
H&E: Hematoxylin and eosin
EB: Embryoid bodies
GEO: Gene Expression Omnibus
