# Supplementary figures and images for: Role of CD133 in human embryonic stem cell proliferation and teratoma formation
Source: Stem Cell Res Ther. 2020 May 27;11:208. doi: 10.1186/s13287-020-01729-0 (PMC7251672; doi:10.1186/s13287-020-01729-0)

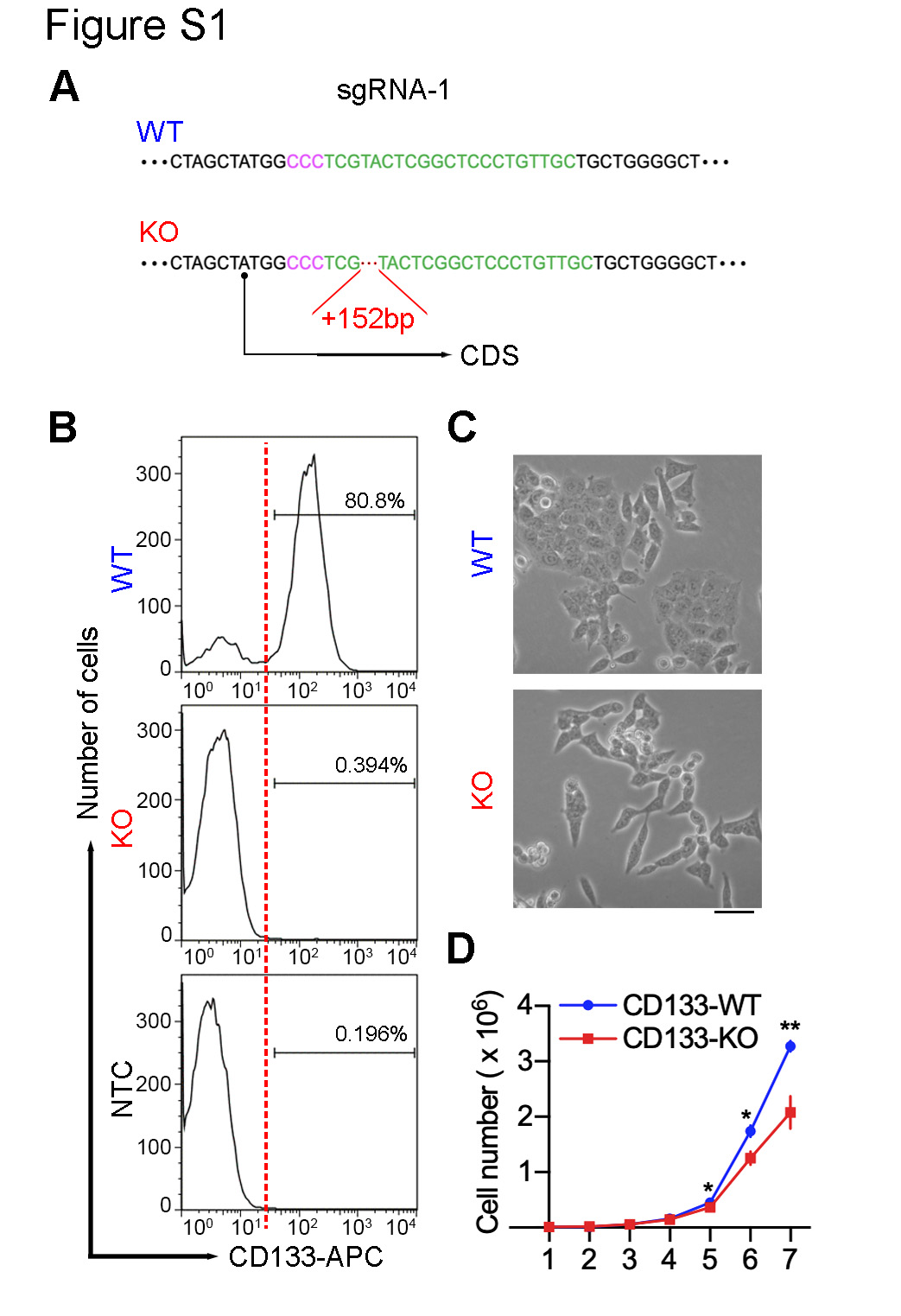

Supplement: Supplementary file 1 — Additional file 1: Figure S1. Knockout of CD133 in HCT116 by CRISPR/Cas9. (A): Sequencing results show a 152bp insertion between the 10th and the 11th base of CDS induced by CRISPR/Cas9 in HCT116 KO clone. (B): Flow cytometry analysis of relative protein level of CD133 in HCT116 WT and KO cell lines. (C): Morphology of CD133 WT and KO HCT116 cells. Scale bar = 50 μm. (D): Growth curves of 7 days show that CD133 KO significantly restrains HCT116 proliferation. Bars indicate mean ± SD (n = 6). [file 13287_2020_1729_MOESM1_ESM.jpg]

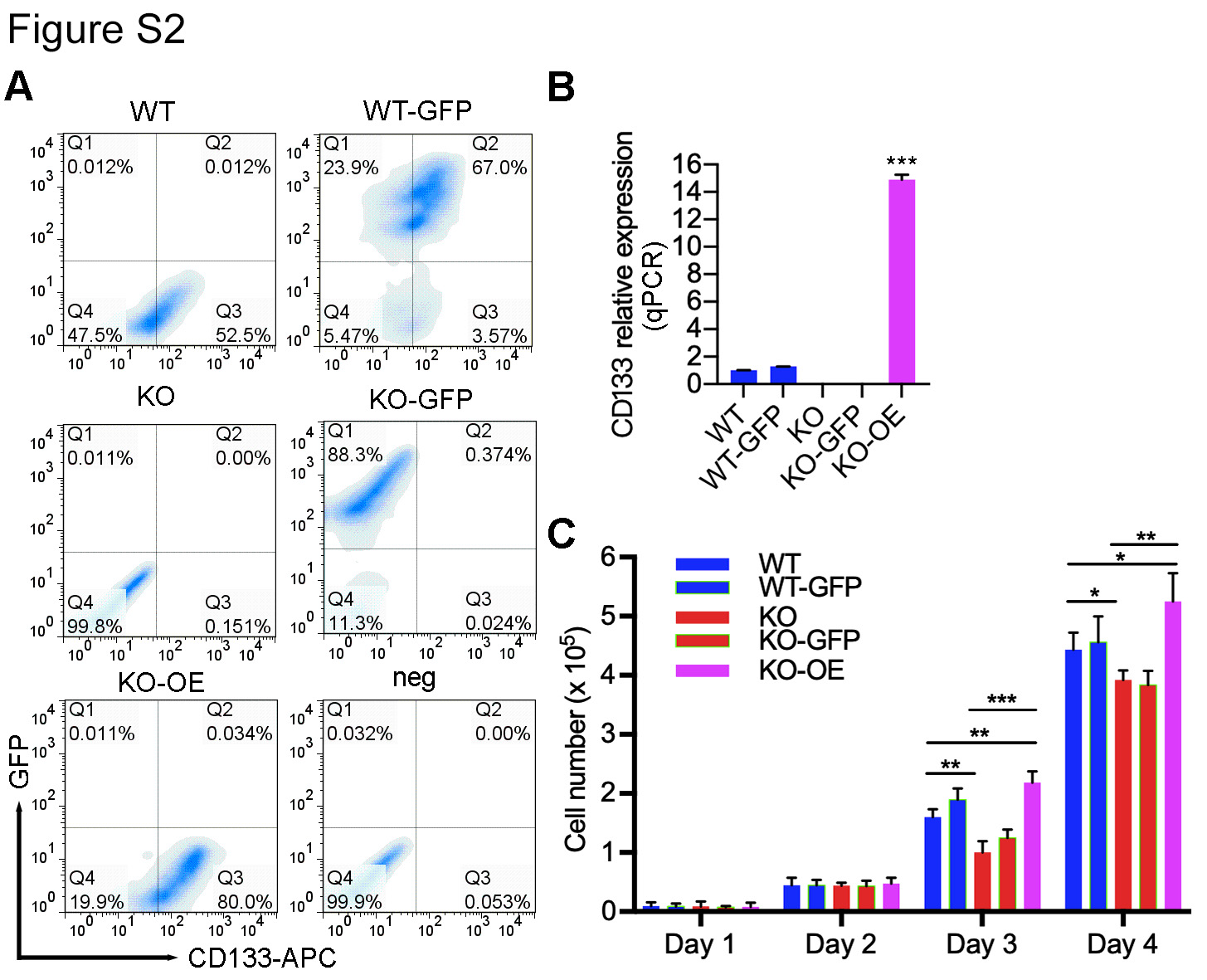

Supplement: Supplementary file 2 — Additional file 2: Figure S2. Rescue of CD133 in CD133 knockout WA26 hESCs. (A): Relative protein level of CD133 and GFP in human cell lines determined by Flow cytometry analysis. (B): Analysis of CD133 mRNA expression of indicated cell lines by RT-qPCR with CD133-qF/R primers (Additional file 4: Table S1). Bars indicate mean ± SD (n≥2). (C):CD133 overexpression significantly rescues the CD133 KO hESC proliferation phenotype by counting cell number. Bars indicate mean ± SD (n = 4). [file 13287_2020_1729_MOESM2_ESM.jpg]

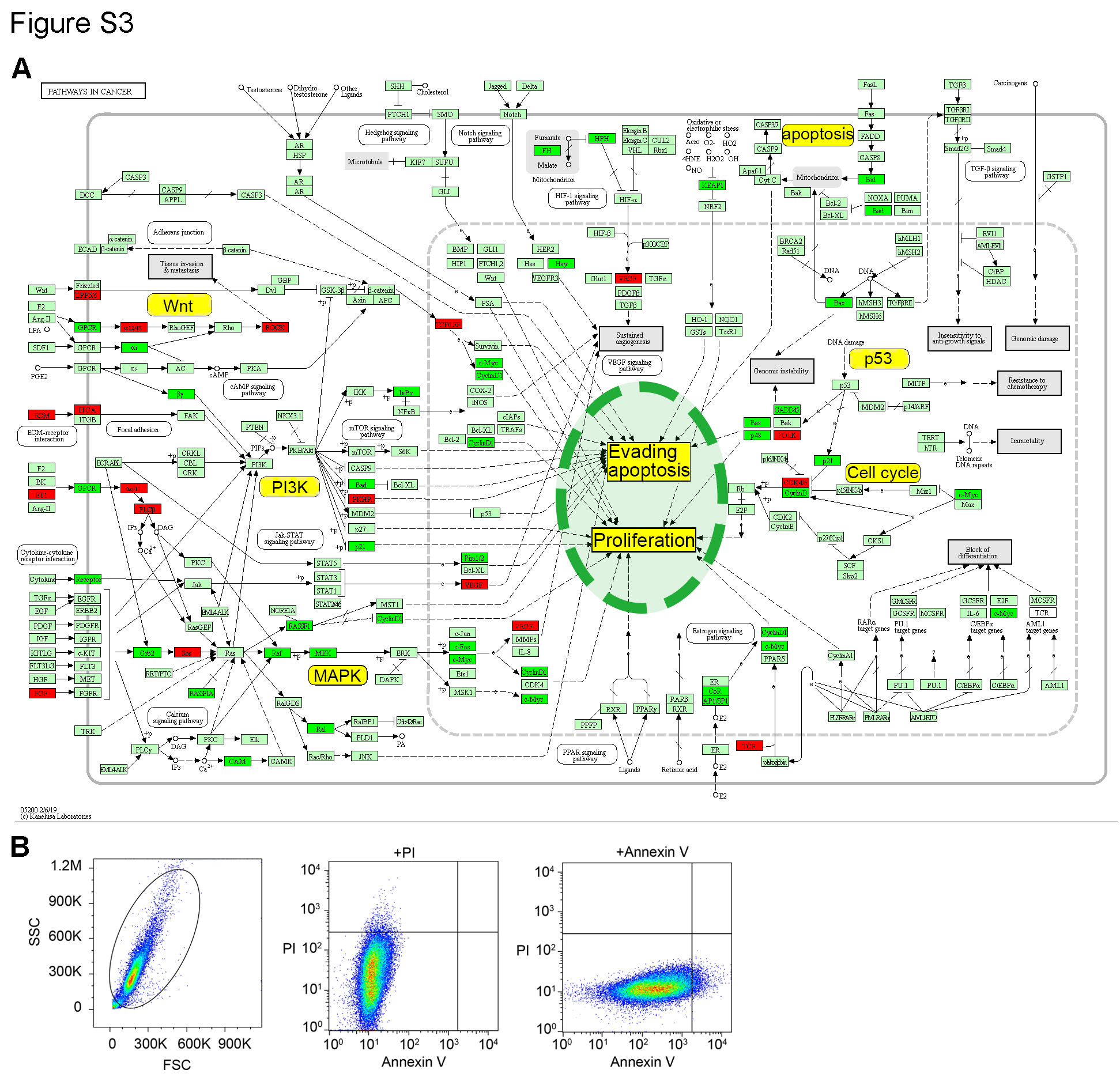

Supplement: Supplementary file 3 — Additional file 3: Figure S3. Overview of general pathways in cancer. (A): All down (dark green) and up (red) regulated genes involved in KEGG cancer pathways, suggesting dysregulation of apoptosis and proliferation. Data analyzed by KEGG Mapper-Search & Color Pathway website. (B): Isotype controls for apoptotic analysis in Fig. 6j. [file 13287_2020_1729_MOESM3_ESM.jpg]
