# Supplementary material for: Role of CD133 in human embryonic stem cell proliferation and teratoma formation
Source: Stem Cell Res Ther. 2020 May 27;11:208. doi: 10.1186/s13287-020-01729-0 (PMC7251672; doi:10.1186/s13287-020-01729-0)
Supplement: Supplementary file 4 — Additional file 4: Table S1. Primers for CAS9, over expression and gene expression analysis. [file 13287_2020_1729_MOESM4_ESM.docx]

**Table S1.** Primers for CAS9, over expression and gene expression analysis.

| Primer name | 5'-3' |
| --- | --- |
| For CRISPR/CAS9 | |
| hCD133-sgRNA-F | CACCGCCAATGGGTCCAGCTTTAT |
| hCD133-sgRNA-R | AAACATAAAGCTGGACCCATTGGC |
| hCD133-sgRNA-1F | CACCGCAACAGGGAGCCGAGTACGA |
| hCD133-sgRNA-1R | AAACTCGTACTCGGCTCCCTGTTGC |
| hCD133-Cas9-F (650) | CATAAAGGGTCTGTGTTCTGAGT |
| hCD133-Cas9-R (650) | CGGCTGTACCACATAGAGAAAG |
| For over expression | |
| hCD133-CDS-F | TTGGCGCGCCGATGGCCCTCGTACTCGG |
| hCD133-CDS-R | GGATCCTCAATGTTGTGATGGGCTTGTC |
| For qPCR | |
| hCD133-1F | CCTCTGGTGGGGTATTTCTTT |
| hCD133-1R | CCAGTTTCCGACTCCTTTTG |
| hCD133-2F [1] | AGTCGGAAACTGGCAGATAGC |
| hCD133-2R | GGTAGTGTTGTACTGGGCCAAT |
| hCD133-qF | AATTATGAGACCCAAGACTCCCATAAAGCT |
| hCD133-qR | GTCATAATCAATTTTGGATTCATATGCCTTCTGTAAGA |
| hGAPDH-F [2] | ACAACTTTGGTATCGTGGAAGG |
| hGAPDH-R | GCCATCACGCCACAGTTTC |
