# Supplementary material for: Role of CD133 in human embryonic stem cell proliferation and teratoma formation
Source: Stem Cell Res Ther. 2020 May 27;11:208. doi: 10.1186/s13287-020-01729-0 (PMC7251672; doi:10.1186/s13287-020-01729-0)
Supplement: Supplementary file 5 — Additional file 5: Table S2. Potential off-target sites (OTs) and primers. [file 13287_2020_1729_MOESM5_ESM.docx]

**Table S2.** Potential off-target sites (OTs) and primers.

| **Off-target NO.** | **Name** | **Primer Sequence 5'-3’** | **Amplicon Length** | **CFD Score** |
| --- | --- | --- | --- | --- |
| hCD133 off-tgt-1-F | mm4_intergenic_IPO11\|RP11-158J3.2_chr5_63251788_F | CCTGTGTCTGCTTCATGAGTAA | 573 | 0.67 |
| hCD133 off-tgt-1-R | mm4_intergenic_IPO11\|RP11-158J3.2_chr5_63251788_R | CCTGAATGATCAGTGAGAGAGTG |  |  |
| hCD133 off-tgt-2-F | mm4_intron_STXBP1_chr9_127679613_F | TACTTGCCATCAGCCTTGTAG | 548 | 0.39 |
| hCD133 off-tgt-2-R | mm4_intron_STXBP1_chr9_127679613_R | TCACTCCCTTCTCTCCTTGT |  |  |
| hCD133 off-tgt-3-F | mm4_intergenic_RP11159D12.11\|VEZF1_chr17_57967190_F | GTCTGCGAAGAGAGAGAGAAAG | 526 | 0.38 |
| hCD133 off-tgt-3-R | mm4_intergenic_RP11-159D12.11\|VEZF1_chr17_57967190_R | CAGCTGTTCCTTATGCCAAATC |  |  |
| hCD133 off-tgt-4-F | mm4_intron_SEMA6A_chr5_116485883_F | TGCCTGTCATTTAGGCACTC | 434 | 0.35 |
| hCD133 off-tgt-4-R | mm4_intron_SEMA6A_chr5_116485883_R | CTGGAAGGCTCTAGTGAGAATTT |  |  |
| hCD133 off-tgt-5-F | mm4_intergenic_AF015720.3\|FKSG68_chr21_35878474_F | CTCAGGAGGTTCTAAGTGGTTATG | 429 | 0.32 |
| hCD133 off-tgt-5-R | mm4_intergenic_AF015720.3\|FKSG68_chr21_35878474_R | TTCTTCCCAGCAGTGCTATTT |  |  |
| hCD133 off-tgt-6-F | mm4_intergenic_RP11-473M14.3\|RP11-542B15.1_chr12_67484445_F | ACCAAGGTAGGGAGGAAGAA | 444 | 0.28 |
| hCD133 off-tgt-6-R | mm4_intergenic_RP11-473M14.3\|RP11-542B15.1_chr12_67484445_R | TGCCAAACTAGGCTGTCTATTC |  |  |
| hCD133 off-tgt-7-F | mm4_exon_IARS2_chr1_220094124_F | CTATTCGAGTTCGGCTGGTG | 627 | 0.25 |
| hCD133 off-tgt-7-R | mm4_exon_IARS2_chr1_220094124_R | CCCAGATTGAGATGTGCAGTAT |  |  |
| hCD133 off-tgt-8-F | mm4_exon_KCNV2_chr9_2718541_F | CAACACCTGACCCACCTTAC | 702 | 0.14 |
| hCD133 off-tgt-8-R | mm4_exon_KCNV2_chr9_2718541_R | GAGGAGCAGACAGACGAATAC |  |  |
| hCD133 off-tgt-9-F | mm4_exon_MAST1_chr19_12867541_F | CACTGCCAGGAAGCTGATTA | 696 | 0.07 |
| hCD133 off-tgt-9-R | mm4_exon_MAST1_chr19_12867541_R | GCAATGTGAATAGGACCTGAGA |  |  |
