# Supplementary material for: Role of CD133 in human embryonic stem cell proliferation and teratoma formation
Source: Stem Cell Res Ther. 2020 May 27;11:208. doi: 10.1186/s13287-020-01729-0 (PMC7251672; doi:10.1186/s13287-020-01729-0)
Supplement: Supplementary file 6 — Additional file 6: Table S3. Pluripotency genes from RNA-seq. [file 13287_2020_1729_MOESM6_ESM.docx]

**Table S3.** Pluripotency genes from RNA-seq.

| Naïve | | | Primed | | | Pluripotency | | |
| --- | --- | --- | --- | --- | --- | --- | --- | --- |
| **Gene name** | **log2FC** | **P (adj)** | **Gene name** | **log2FC** | **P (adj)** | **Gene name** | **log2FC** | **P (adj)** |
| TDGF1 | -0.6763 | 0.0008 | THY1 | 0.7099 | 0.0032 | POU5F1 | 0.8516 | 0.0016 |
| DPPA2 | -0.4861 | 0.0979 | ZIC5 | -0.4721 | 0.2080 | NANOG | 0.0098 | 0.9797 |
| GDF3 | -0.0169 | 0.9667 | DUSP6 | 0.9184 | 0.0002 | SOX2 | -0.5100 | 0.0722 |
| LEFTY2 | 0.2770 | 0.6589 | ZIC2 | 0.0323 | 0.9412 | LIN28A | -0.4168 | 0.0629 |
| NODAL | 0.6971 | 0.0385 | FGF2 | -0.9845 | 0.0011 |  |  |  |
| TBX3 | -0.1665 | NA | OTX2 | -0.8177 | 0.0393 |  |  |  |
| DPPA5 | -1.0684 | 0.0120 | DNMT3B | -0.3482 | 0.1774 |  |  |  |
| IL6ST | -0.4631 | 0.3354 | NODAL | 0.6971 | 0.0385 |  |  |  |
| DPPA3 | -0.2776 | 0.6537 | POU3F1 | 0.0128 | NA |  |  |  |
| PRDM14 | 0.5511 | 0.1728 | SOX11 | -0.7658 | 0.0011 |  |  |  |
| TFCP2L1 | -0.3330 | NA | SFRP2 | 1.2283 | 0.0000 |  |  |  |
| KLF4 | 0.3839 | NA | SALL2 | -0.4724 | 0.0877 |  |  |  |
| KLF5 | 0.1244 | NA |  |  |  |  |  |  |
| ZFP42 | -0.1984 | 0.5178 |  |  |  |  |  |  |
| TBX3 | -0.1665 | NA |  |  |  |  |  |  |
| ESRRB | -0.1210 | NA |  |  |  |  |  |  |
| DNMT3L | -0.0587 | NA |  |  |  |  |  |  |
| UTF1 | 0.5710 | 0.2791 |  |  |  |  |  |  |
| SOX15 | 0.9423 | 0.0100 |  |  |  |  |  |  |

Significant *P* values were colored for down (green) and up (red) regulated genes. Genes were collected according to previous publications [3-6].
