# Supplementary material for: Role of CD133 in human embryonic stem cell proliferation and teratoma formation
Source: Stem Cell Res Ther. 2020 May 27;11:208. doi: 10.1186/s13287-020-01729-0 (PMC7251672; doi:10.1186/s13287-020-01729-0)
Supplement: Supplementary file 7 — Additional file 7: Table S4. Genes related to embryonic germ layers from RNA-seq. [file 13287_2020_1729_MOESM7_ESM.docx]

**Table S4.** Genes related to embryonic germ layers from RNA-seq.

| **Category** | | **Gene name** | **log2FC** | **P (adj)** |
| --- | --- | --- | --- | --- |
| ectoderm | | GATA3 | -0.0360317 | NA |
|  |  | TFAP2C | -0.3799279 | 0.25411422 |
|  |  | ALCAM | -0.4742323 | 0.18793925 |
|  |  | OLFM1 | 0.13467372 | 0.76416173 |
| mesoderm | | GATA6 | -0.0717297 | NA |
| mesendoderm | | T | -0.1629639 | NA |
|  |  | FGF4 | -0.1057842 | NA |
|  |  | LEFTY1 | 0.97969863 | NA |
|  |  | EOMES | -0.3569287 | NA |
|  |  | GSC | 0.81567216 | NA |
|  |  | GATA4 | -0.20385 | NA |
| endoderm | | KLF8 | -0.1130566 | 0.85946551 |
| starting point of differentiation | | SOX2 | -0.5099657 | 0.07217245 |
|  |  | PRDM14 | 0.55107869 | 0.17283882 |
|  |  | ZIC2 | 0.03229521 | 0.94116753 |
| middle stage of differentiation | endothelial cell | TFAP2A | -0.1000807 | NA |
|  | muscle cell | MEF2C | -0.309467 | NA |
|  | stromal cell | PAX3 | -0.234957 | NA |
|  |  | SALL1 | -0.2886811 | 0.42548001 |
|  | liver cell | MEIS2 | -0.1306448 | NA |
|  | epithelial | ARID5B | -0.4567049 | 0.29774458 |
|  |  | CASZ1 | 0.00190644 | NA |
|  | neural | PKNOX2 | 0.08513438 | NA |
|  |  | TBX3 | -0.1665346 | NA |
| terminal stage of differentiation | endothelial cell | GATA2 | 0.10107969 | NA |
|  |  | TAL1 | -0.4989969 | NA |
|  | muscle cell | ZFHX3 | -0.2212046 | 0.72821465 |
|  | stromal cell | KLF6 | 0.39174942 | 0.38077198 |
|  |  | MAF | -0.05412 | 0.93179401 |
|  | liver cell | EOMES | -0.3569287 | NA |
|  |  | SOX7 | 0.0170218 | NA |
|  | epithelial | SOX9 | -0.1002606 | 0.88503329 |
|  |  | FOXP1 | -0.2007149 | 0.67973601 |
|  | Neural | PAX3 | -0.234957 | NA |

Genes were collected according to previous publications [7-9].

**References**

1 Liu J, Mao Z, Huang J, Xie S, Liu T,Mao Z. Blocking the NOTCH pathway can inhibit the growth of CD133-positive A549 cells and sensitize to chemotherapy. Biochem Biophys Res Commun 2014; 444: 670-5.

2 Ross J, Busch J, Mintz E, Ng D, Stanley A, Brafman D et al. A rare human syndrome provides genetic evidence that WNT signaling is required for reprogramming of fibroblasts to induced pluripotent stem cells. Cell Rep 2014; 9: 1770-80.

3 Collier AJ, Panula SP, Schell JP, Chovanec P, Plaza Reyes A, Petropoulos S et al. Comprehensive Cell Surface Protein Profiling Identifies Specific Markers of Human Naive and Primed Pluripotent States. Cell Stem Cell 2017; 20: 874-90 e7.

4 Gafni O, Weinberger L, Mansour AA, Manor YS, Chomsky E, Ben-Yosef D et al. Derivation of novel human ground state naive pluripotent stem cells. Nature 2013; 504: 282-6.

5 Heinaniemi M, Nykter M, Kramer R, Wienecke-Baldacchino A, Sinkkonen L, Zhou JX et al. Gene-pair expression signatures reveal lineage control. Nat Methods 2013; 10: 577-83.

6 Nakamura T, Okamoto I, Sasaki K, Yabuta Y, Iwatani C, Tsuchiya H et al. A developmental coordinate of pluripotency among mice, monkeys and humans. Nature 2016; 537: 57-62.

7 Chu LF, Leng N, Zhang J, Hou Z, Mamott D, Vereide DT et al. Single-cell RNA-seq reveals novel regulators of human embryonic stem cell differentiation to definitive endoderm. Genome Biol 2016; 17: 173.

8 Han X, Chen H, Huang D, Chen H, Fei L, Cheng C et al. Mapping human pluripotent stem cell differentiation pathways using high throughput single-cell RNA-sequencing. Genome Biol 2018; 19: 47.

9 Li L, Wang Y, Torkelson JL, Shankar G, Pattison JM, Zhen HH et al. TFAP2C- and p63-Dependent Networks Sequentially Rearrange Chromatin Landscapes to Drive Human Epidermal Lineage Commitment. Cell Stem Cell 2019; 24: 271-84 e8.
